# Cytomegalovirus Hepatitis in an Immunocompetent Patient: A Cause of Fever of Unknown Origin

**DOI:** 10.7759/cureus.10745

**Published:** 2020-10-01

**Authors:** Saud Bin Abdul Sattar, Muhammad Adnan Haider, Zeeshan Zia, Muhammad Niazi, Qasim Z Iqbal

**Affiliations:** 1 Internal Medicine, Northwell Health, New York City, USA; 2 Internal Medicine, Allama Iqbal Medical College/Jinnah Hospital, Lahore, PAK

**Keywords:** hepatitis, cytomegalovirus (cmv), antiviral therapy, internal medicine (general medicine)

## Abstract

Acute hepatitis is most often self-resolving and a benign condition that rarely requires any anti-viral drugs. In immunocompromised patients (HIV-infected patients and transplant recipients), the morbidity and mortality associated with cytomegalovirus (CMV) infection have been extensively reported in the medical literature. We are describing a rare case of acute severe cytomegalovirus hepatitis in an immunocompetent host. In immunocompetent individuals, in most cases, it causes a subclinical infection and hence doesn't require an anti-viral agent for treatment. Our patient was unique because it presented with clinically severe hepatitis and was uncharacteristically treated with the use of antiviral medications.

## Introduction

Cytomegalovirus (CMV) belongs to the herpes family of viruses. It is a very common pathogen in humans. The seroprevalence for CMV in the world hence is 60-100% but the severity of symptoms varies among different individuals. CMV infection in most cases presents as a subclinical infection. However, in immunocompromised hosts, primary CMV infection, reactivation, and re-infection are all common. In these patients, it is unfortunately associated with both significant morbidity and mortality [1, 2]. Fortunately though, in the immunocompetent adult, primary CMV infection is in most cases asymptomatic. It can occasionally, however, result in a mononucleosis syndrome [3]. More importantly, CMV infection in immunocompetent hosts can very rarely lead to severe organ-specific complications. In these patients, hepatitis despite being rare and uncommon can hence be seen [4]. We describe an interesting clinical case of CMV hepatitis as seen in an immunocompetent host. This case was also fascinating as it was severe enough to require treatment with an antiviral agent. [5]

## Case presentation

A 59-year-old male with a pertinent medical history of dyslipidemia recently treated with rosuvastatin presented to the emergency department (ED) with the chief complaint of fever for the last three weeks. The fever was high grade in intensity, ranging from 100.5°F to 102.2°F, gradual in onset, intermittent in nature, and progressively worsening. It was associated with fatigue, chills, and diaphoresis. On reviewing other systems he further mentioned an unintentional weight loss of 10 pounds during the last three months. He denied any nausea, abdominal pain, tenderness, diarrhea, constipation, drug abuse history, chest pain, shortness of breath, cough, palpitations, night sweats, weakness, muscle aches, joint pain, or any other complaints. He had no recent travel history outside the US over the last one year. The only medical disease he had was hypercholesterolemia for which has was started on 10 mg rosuvastatin tablet six months ago but was advised by his primary care doctor to stop it a month ago due to mild transaminitis on lab work as an outpatient. He had no previous surgical history and the family history was not pertinent.

On arrival at the ED, the patient was febrile with a temperature of 101°F and tachycardia with a heart rate of 121 beats per min; all other vital signs were within normal limits. On physical examination, he was anicteric, had no abdominal palpable mass or tenderness with normal bowel sounds. The rest of the physical examination was also unremarkable. On investigation, his blood work was within normal range except for an increase in the levels of the liver enzyme with alanine aminotransferase (ALT) at 187 U/L (n=0-41 U/L) and aspartate aminotransferase (AST) at 152 U/L (n=0-41 U/L). Complete blood count (CBC) with differential and serum electrolytes were also normal. There were no abnormalities in bilirubin and alkaline phosphatase. The chest X-ray was unremarkable. The initial CT scan of the abdomen showed mild splenomegaly, a small amount of pelvic ascites, and a fatty liver. The patient was admitted with suspicion of sepsis from an unknown cause. The symptoms of the patient, hepatic-steatosis with an increase in both ALT and AST suggested hepatocellular damage, most likely hepatitis (Table 1).

**Table 1:** Laboratory investigations favoring hepatitis

Investigations	At admission	Peak value during hospital stay
Alanine aminotransferase, U/L	187	210
Aspartate aminotransferase, U/L	152	162
Total bilirubin, mg/dl	1.0	1.0
Alkaline phosphatase, U/L	107	115

Cultures of his blood and urine were done on admission and initial management with intravenous fluids followed by a dose of ceftriaxone 1000 mg along with Tylenol 650 mg was started in ED. The patient remained stable during his inpatient stay with the exception of daily spikes in temperature as high as 103.1°F around early evening. The urine and blood cultures later came back negative for any microbial growth. The investigation continued with a toxicological profile, acute hepatitis panel for hepatitis A, B, and C viruses antigens and antibodies, serum acetaminophen level, urine copper and serum ceruloplasmin, as well as an antinuclear antibodies (ANA) test with an autoimmune profile, and all the results came out negative. The patient’s serum inflammatory markers were high, including ferritin 2888 ng/mL (n=30-400 ng/mL) along with transaminitis and he was continuously spiking high-grade fever daily afternoon with progressive fatigue during his seven-day hospital course. After seven days of investigation and evaluation by infectious disease and heme-oncologist, he has declared as a case of fever of unknown origin, and a bone marrow biopsy was scheduled to rule out hemophagocytic lymphohistiocytosis (HLH). On day eight in the hospital, a CBC was performed with peripheral smear and flow cytometry, revealing atypical lymphocytosis with no evidence of clonality. Later on the same day, the serology for CMV came out positive with the polymerase chain reaction (PCR) showing a value of 1692 with CMV PCR log of 3.23 (normal <0.00 Log IU/ml) indicating a disseminated cytomegalovirus infection (Table 2). Given that all other causes of hepatitis were excluded, it was safely concluded that the patient had an acute CMV hepatitis.

**Table 2:** The investigations in our patients pointing towards a cytomegalovirus infection PCR: polymerase chain reaction

Finding	Results
Absolute lymphocytes, cells/uL (n=850-3900 cells/uL)	7203
Reactive lymphocyte % (n= 0-5 %)	16.8
Cytomegalovirus PCR log (n < 0.00 log IU/mL)	3.23
Cytomegalovirus PCR	1692

The decision to treat this acute viral hepatitis with antiviral therapy was made due to its severe course. Treatment was started with valganciclovir 900 mg intravenously twice a day in the hospital and upon discharge shifting it to oral 900 mg every 12 hours for a total of 14 days. The patient symptomatically improved and became fully functional thereafter.

## Discussion

The prevalence of CMV infection is very high, but this infection is usually self-limiting. In immunocompromised patients, a wide range of severe clinical symptoms have been reported in medical literature but little attention has been given to CMV infection in the immunocompetent. Mostly, CMV infection is mild in such immunocompetent patients. We describe a case of an elderly person who presented with severe CMV associated hepatitis. Hepatitis is a common disease entity caused by a multitude of processes. A wide range of factors can cause hepatitis like drugs (acetaminophen and many other drugs) [6], hemochromatosis, Wilson’s disease, primary biliary cirrhosis, non-alcoholic steatotic hepatitis, and most commonly the viral illness with hepatitis A, B, and C. However, CMV associated viral hepatitis have been reported in immunocompromised hosts or in liver transplant recipients. The incidence of CMV hepatitis in such patients ranges from 2% to 34% [7, 8]. The viral burden is compounded by a series of factors, including immunosuppressive regimens, the serological status of a positive donor, and a high viral load in peripheral blood.

The first case of CMV associated hepatitis in immunocompetent patients was reported by Lamb and Stern [9]. Although still infrequent, the sporadic cases of CMV-induced hepatitis hardly result in fulminant liver failure [10, 11]. The disease usually presents with a moderate increase in bilirubin and alkaline phosphatase (ALP) and symptoms spontaneously resolve in a few days in such patients. AST and ALT are the most altered enzymes but don’t exceed above fivefold of the normal reference value. These findings are self-limiting with transaminase enzymes becoming normal in a few weeks [12]. Although extremely rare, CMV associated acute fulminant hepatic failures requiring emergency liver transplant have been described in the past [13, 14].

In our case, the patient presented with a high-grade fever that failed to resolve with symptomatic treatment. CMV serology came out positive and all other causes of hepatitis were ruled out. The decision was made to treat this patient with valganciclovir due to the severity of the disease and the same was done in formerly reported such cases of CMV associated severe hepatitis in the medical literature [5]. Importantly, without the serology for CMV, it would have been impossible to determine the cause, indicating its importance.

## Conclusions

We present a case of hepatitis caused by cytomegalovirus in an immunocompetent male patient. This is one of the few cases of CMV-induced hepatitis causing sepsis in an immunocompetent patient, requiring hospitalization and treatment with an antiviral agent. This report emphasizes the importance of investigating CMV as a causative agent irrespective of the immune status in a patient with hepatitis. This is especially important when clinical signs and history warrant such testing. The treatment of this illness is only warranted if the patient continues to worsen despite symptomatic treatment. This is different from other common causes of acute viral hepatitis, e.g., hepatitis A and E, in which we do not treat acute hepatitis with any antiviral agent.

## References

[1] Vancíková Z, Dvorák P (2001). Cytomegalovirus infection in immunocompetent and immunocompromised individuals--a review. Curr Drug Targets Immune Endocr Metabol Disord.

[2] Giroud O, Meier P, San Millán D (2010). Severe CMV infection: not only in immunocompromised patients. Rev Med Suisse.

[3] Wreghitt TG, Teare EL, Sule O, Devi R, Rice P (2003). Cytomegalovirus infection in immunocompetent patients. Clin Infect Dis.

[4] Mamun-Al-Mahtab Mamun-Al-Mahtab, Rahman S, Khan M (2009). Acute cytomegalovirus hepatitis in immunocompetent host. Kathmandu Univ Med J.

[5] Padala SK, Kumar A, Padala S (2014). Fulminant cytomegalovirus myocarditis in an immunocompetent host: resolution with oral valganciclovir. Tex Heart Inst J.

[6] Wang K, Huang YS, Deng JF (1999). Characteristics and risk factors of acetaminophen-induced hepatitis in Taiwan. Chin Med J.

[7] Azevedo LS, Pierrotti LC, Abdala E (2015). Cytomegalovirus infection in transplant recipients. Clinics (Sao Paulo).

[8] Kanj SS, Sharara AI, Clavien PA, Hamilton JD (1996). Cytomegalovirus infection following liver transplantation: review of the literature. Clin Infect Dis.

[9] Lamb SG, Stern H (1966). Cytomegalovirus mononucleosis with jaundice as presenting sign. Lancet.

[10] Rafailidis PI, Mourtzoukou EG, Varbobitis IC, Falagas ME (2008). Severe cytomegalovirus infection in apparently immunocompetent patients: a systematic review. Virol J.

[11] Azad AK, Ahmed T, Chowdhury AJ (2008). Cytomegalovirus induced hepatitis in an immunocompetent host. Mymensingh Med J.

[12] Bonnet F, Neau D, Viallard JF (2001). Clinical and laboratory findings of cytomegalovirus infection in 115 hospitalized non-immunocompromised adults. Ann Med Interne (Paris).

[13] Kano Y, Shiohara T (2000). Current understanding of cytomegalovirus infection in immunocompetent individuals. J Dermatol Sci.

[14] Shusterman NH, Frauenhoffer C, Kinsey MD (1978). Fatal massive hepatic necrosis in cytomegalovirus mononucleosis. Ann Intern Med.

